# Thermophysical properties of tetrabutylammonium chloride, paraffin and fatty acids for thermal energy applications†

**DOI:** 10.1039/d4ra03782k

**Published:** 2024-08-19

**Authors:** Tomás Costa, Yolanda Sanchez-Vicente, Zili Yang, Lee A. Stevens, Fabio de S. Dias, Sol-Carolina Costa Pereira

**Affiliations:** a Department of Mechanical and Construction Engineering, Northumbria University Newcastle Upon Tyne NE1 8ST UK Yolanda.vicente@northumbria.ac.uk; b University of Nottingham, Low Carbon Energy and Resources Technologies Group, Faculty of Engineering Energy Technologies Building, Triumph Road Nottingham NG7 2TU UK; c Universidade Federal da Bahia, Instituto de Química, Departamento de Química Analítica 40170-280 Salvador Bahia Brazil; d School of Computing, Engineering & the Built Environment, Edinburgh Napier University EH10 5DT UK

## Abstract

Investigating the thermophysical properties of substances is crucial for using them as phase change materials (PCMs) and heat transfer fluids (HTFs) in thermal energy applications. In this study, the thermophysical properties of three medium-temperature PCMs (around 338 K) and one ionic liquid, tetrabutylammonium chloride ([N_4444_^+^][Cl^−^]), were evaluated and compared. The commercial PCMs were two fatty acids (OM65 and stearic acid) and one paraffin (RT64HC). The characterised thermophysical properties were the viscosity, density, phase change temperatures, melting and solidification enthalpies, and thermal conductivity for the solid and liquid phases. The uncertainties for each property were calculated, and two empirical equations were obtained from the correlation of viscosity and thermal conductivity data along isotherms. This paper also compared the thermophysical properties of commercial PCMs and HTFs against the ionic liquid, discussing the potential use of the ionic liquid as a thermal energy storage material and HTFs.

## Abstract 2

Viscosities, densities, phase change enthalpies and thermal conductivities of OM65, stearic acid, RT64HC and tetrabutylammonium chloride for thermal application.

## Graphical abstract

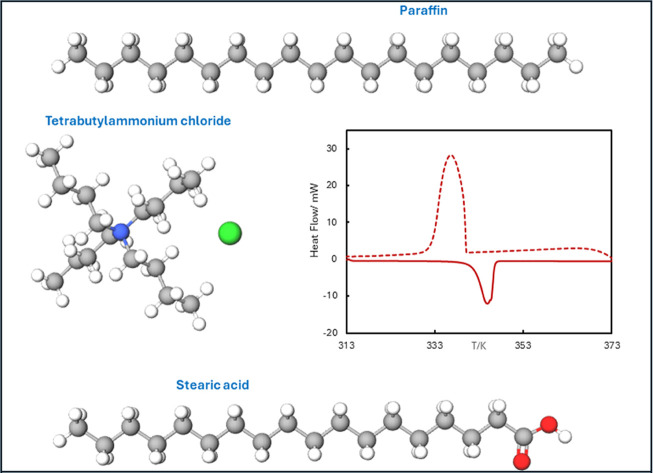

## 1. Introduction

Global thermal end-uses (space and water heating) accounted for 53% of the total energy consumption in buildings in 2020. Considering the increasing demand, integrating thermal energy storage (TES) is crucial in transitioning towards a net-zero building and a carbon-neutral economy. Phase change materials (PCMs) and effective novel heat transfer fluids (HTFs)^1–4^ are relevant in developing highly efficient TES systems. Low-temperature PCMs are especially suitable for TES in buildings, as they can be integrated into domestic heating, water tanks and air-conditioning equipment. Among medium-temperature PCMs (between 313 and 573 K), paraffin waxes and fatty acids have been extensively studied due to their low cost, chemical stability, heat storage capacity, and non-toxic effects.^5^ Nevertheless, its application is still limited due to its low thermal conductivity and flammability for paraffin. On the other hand, common HTFs like aliphatic and aromatic hydrocarbons, polyhydric alcohols, and organosilicon fluids also come with drawbacks such as high vapor pressure and flammability for organic HTFs, and higher costs. Therefore, more research, development and innovation in materials science and system integration are required to deploy this technology.

Ionic liquids (ILs) are a group of molten salts with a wide temperature range for the liquid phase, high heat capacity, high density, high thermal conductivity and chemical stability, low vapour pressure, and non-harmfulness.^6,7^ ILs can be designed by selecting the appropriate cations (such as imidazolium, pyridinium, quaternary ammonium and phosphonium ions), anions (such as halogens, nitrate, acetate) and alkyl side chain groups.^8,9^ In 2001, the first studies on synthesis, characterisations and evaluations of the feasibility of ILs as liquid TES,^6,10^ showed that ILs can have superior heat-transfer properties compared to several commercial heat-transfer fluids. Zhu *et al.*^8^ measured the melting point (range from 303 K to 373 K), the heat of fusion and heat capacities for a series of imidazolium-based ILs. Their results indicated that long-chain alkylimidazolium bromides could be used as PCMs with melting enthalpies above 120 kJ kg^−1^. Bhatt and Gohil^11^ synthesised and characterised eight ILs based on tetrabutylammonium cation and inorganic anions (melting point range from 317 K to 383 K). They recommended ILs containing nitrate, nitrite and iodate anions for thermal applications from 223 K to 383 K such as a solar cooker.^12^ Escribà *et al.*^13^ investigated ILs (diimidazol-1-ium esters) prepared from wastes, crude glycerol and carboxylic acid as bio-based PCM. They found that some ILs presented melting enthalpies about 300 kJ kg^−1^ much higher than commercial PCMs. Bendová *et al.*^14^ measured the heat capacity of two ILs with a 1-hexadecyl-3-methylimidazolium cation and different anions, chloride and saccharinate, with melting temperatures of 324 K to 338 K and melting enthalpies close to or higher than 100 kJ kg^−1^, demonstrating their potential as PCMs for latent heat storage. Vijayraghavan *et al.*^9^ investigated the promising properties of protic organic salts as ILs, based on guanidinium cation showing that has a range for both enthalpy of fusion Δ*H*_f_ (69–190 kJ kg^−1^) and *T*_m_ (373–500.95 K). They attributed the high melting coupled with high melting enthalpy to the presential of extensive hydrogen bonding in the system. They suggest these salts have good potential for thermal-storage media for low-to-medium temperatures such as organic Rankine cycles.^15^ Valkenburg *et al.* evaluate the thermophysical properties of three ionic liquids based on imidazolium cation for application as HTFs for solar collectors.^10,16^ Fabre *et al.* review the main thermophysical properties of ionic liquids for their applications to HTFs.^1^ All these articles support the potential of ionic liquid as PCMs and HTFs for thermal energy application. However, the commercial applications of IL are still limited due to the high cost and high viscosity.

The thermophysical properties of PCM and HTFs are required to design and develop effective thermal energy applications such as sizing thermal storages or designing heat exchangers. Several reviews show that thermophysical properties for ionic liquids are available in the literature but unfortunately, are restricted to some fluids and limited properties, and usually, lack of information about the accuracy of the data, which can contribute to design errors.^17^ Furthermore, few studies determined the melting enthalpy and thermal conductivity of the ionic liquids.^1,18^ The available literature lacks of a systematic comparison of the thermophysical properties of medium-temperature ionic liquids against the commercially organic PCMs or HTFs, for thermal energy applications.

In this context, this paper focuses on reporting and systematically comparing the thermophysical properties of three commercially medium-temperature organic PCMs, two fatty acids (OM65 and stearic acid), one paraffin (RT64HC) and one ionic liquid, tetrabutylammonium chloride. The ionic liquid selected in this study is tetrabutylammonium chloride ([N_4444_^+^][Cl^−^]) ammonium-based ionic liquid, with a similar melting temperature to the reference organic PCMs selected, around 343 K and because it was found that ammonium-based ILs usually exhibit higher thermal conductivities,^1^ electrochemical cathodic stabilities and low viscosities^16^ in comparison to other ionic liquids. To the best of our knowledge, no thermophysical property data were available for the [N_4444_^+^][Cl^−^] ionic liquid. Furthermore, it can be obtained from neutralising renewable sources such as amino acids.^19^ A comparison between the properties of the different materials is discussed and the potential applications of the [N_4444_^+^][Cl^−^].

## 2. Materials and characterisation procedures

### 2.1 Materials

**Fig. 1:**
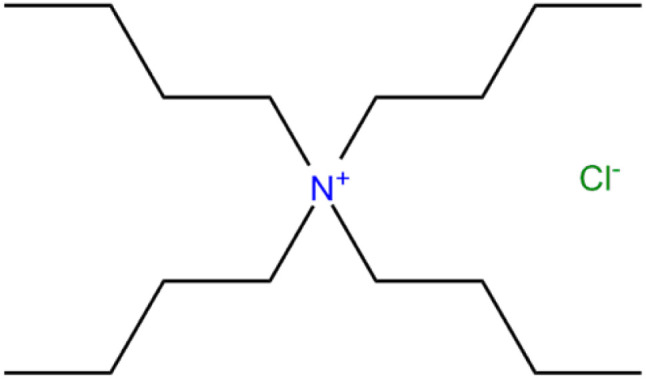
Structure of tetrabutylammonium chloride.^20^

Table 1 describes the phase change materials employed in this study. The tetrabutylammonium chloride, was kept under vacuum in presence of silica gel to avoid exposure to air since ionic liquids tend to absorb moisture. The structure of tetrabutylammonium chloride, [N_4444_^+^][Cl^−^] is shown in Fig. 1. No other analysis or purification was attempted.

**Table 1:** Phase change materials used in this work and sources. Purity content according to manufacture

Name	Type	CAS no.	Chemical formula	Purity	Sources
Tetrabutylammonium chloride, [N_4444_^+^][Cl^−^]	Ionic liquids	1112-67-0	C_16_H_36_ClN	95%	BLD Pharm
				<7% H_2_O	
savE OM65	Fatty acids	68440-15-3	Mixture of fatty acids		PLUSS
Rubitherm RT64HC	Paraffin	8002-74-2	Mixture of paraffins		Rubitherm
Stearic acid	Fatty acid	57-11-4	C_18_H_36_O_2_	<95%	Sigma-Aldrich

In this work, the combined standard uncertainty for all the reported data was calculated following the guidelines described in the GUM^21^ and it is described in the ESI,† as well as the equipment calibration. The experimental mean values and estimated uncertainties are reported for all the properties.

### 2.2 Viscosity

Viscosity measurements were performed using a Brookfield DV-E digital viscometer attached to a thermoset accessory to control the temperature with an uncertainty of ±1 K from 293 K to 573 K. The spindle was SC4-18, designed to measure viscosity from 3 mPa s to 10 000 mPa s. The measurement procedure was as follows: 9 mg of material was placed in the viscometer; temperature was increased until the melting temperature. The spindle was submerged in the melted sample, and the measurements were taken after 20 min to ensure thermal stability from the melting temperature to about 110 °C. At each temperature, 5 viscosity measurements were taken for 15 minutes. Three samples were measured for each substance. The repeatability of the viscosity measurements was lower than 2%.

### 2.3 Density

The principle of Archimedes was applied to calculate the solid density of the samples. The procedure is detailed in the BS EN ISO 1183-1 standard. The Ohaus density determination kit and a Fisherbrands balance with the uncertainty of (0.0001 g) were used. The temperature uncertainty was ±0.5 K. Samples were first weighed in the air (*m*_air_) and then in ethanol (*m*_E_) at temperatures from 287 to 296 K. The density (*ρ*) of the samples was provided by the following equation:1
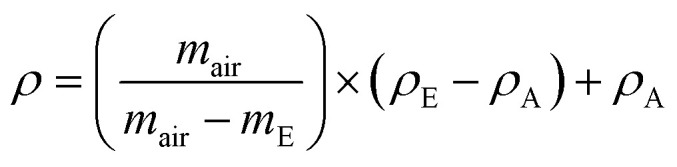
where, *ρ*_E_ and *ρ*_A_ is the density (in kg m^−3^) of ethanol and air respectively and 0.1 MPa. Weighing the dry sample before and after immersion in ethanol indicated that moisture uptake was negligible during the measurement. The density was measured for at least three samples. Density measurements of [N_4444_^+^][Cl^−^] were not conducted due to its solubility in ethanol.

### 2.4 Thermogravimetric analysis

**Fig. 2:**
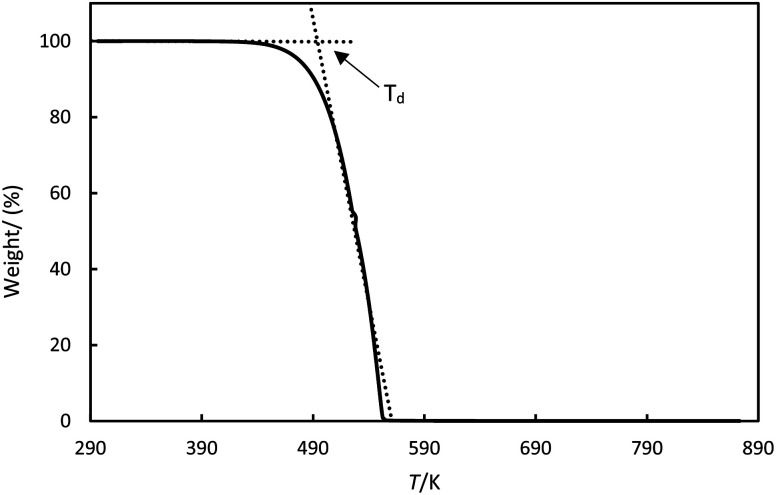
Thermogravimetric analysis for stearic acid illustrates how the thermal decomposition temperature, *T*_d_, was determined.

Thermogravimetric analysis (TGA) evaluate the thermal stability. A TA Instruments Q-550 was used, the samples were heated up to 800 K at 10 K min^−1^, 5 K min^−1^ and 1 K min^−1^ under nitrogen flow to prevent oxidation during the measurement. About 50 mg of sample was placed on platinum crucibles of 100 μL. The effects of the changes in gas viscosity, density and buoyancy were corrected by measuring the response of an empty platinum crucible. The mass/weight precision in the equipment is about ±0.01%. The dynamic thermal decomposition temperatures (*T*_d_) were determined from the measurements using the intersection of the baseline and the tangent line derived from the decomposition curve, as shown in Fig. 2.

It was observed that the *T*_d_ depend on the heating rate. The TGA diagrams are presented in the ESI.† Therefore, we reported the values at the lowest heating rate of 1 K min^−1^ to ensure thermal equilibrium.

### 2.5 Phase transition temperatures and enthalpies

The phase transition temperatures, melting (*T*_m_), and solidification temperatures (*T*_s_) and the melting and solidification enthalpies were investigated using a Heat Flux Differential Scanning Calorimeter (hf-DSC) technique.

#### 2.5.1 Measurement principle

**Fig. 3:**
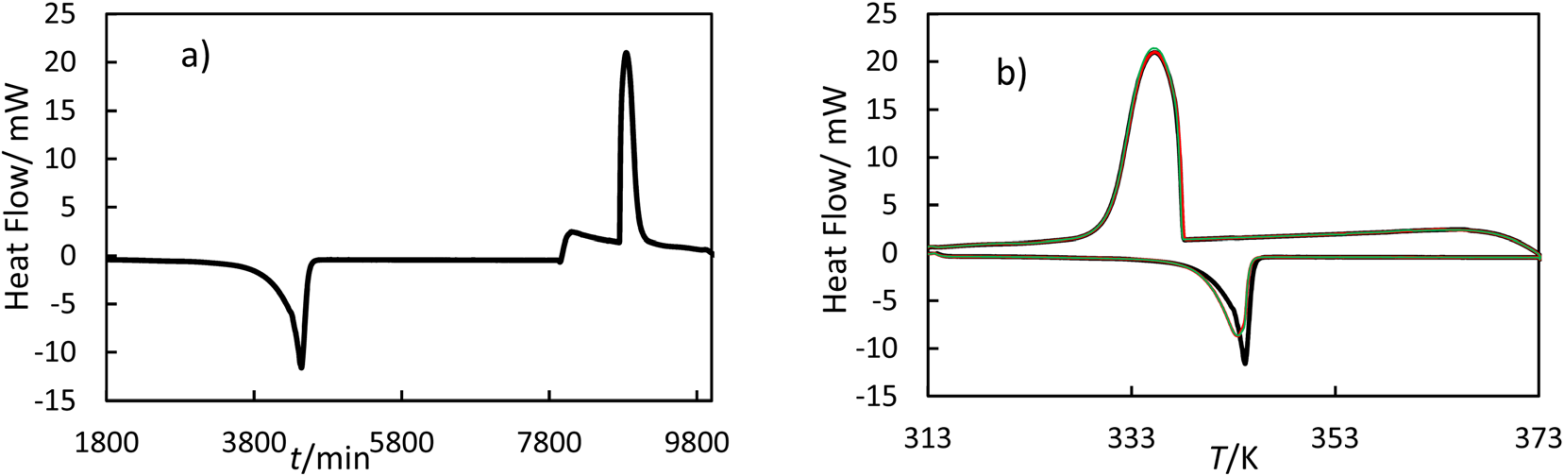
Thermogram of OM65 at 0.5 K min^−1^ obtained by hf-DSC; (a) heat flow *versus* time for one cycle and (b) heat flow *versus* temperature for three cycles: black line, cycle 1; red line, cycle 2; and green line cycle 3.

The hf-DSC measures the difference in heat flow between the sample and empty crucible. Fig. 3 shows the heat flow as a function of time and as a function of temperature for OM65 for the heating and cooling ramps. Fig. 3b shows the melting and solidification processes of the OM65, corresponding to an endothermic and an exothermic peak, respectively. The DSC lines for the heating and cooling ramps overlap across the three cycles, indicating minimal hysteresis.

**Fig. 4:**
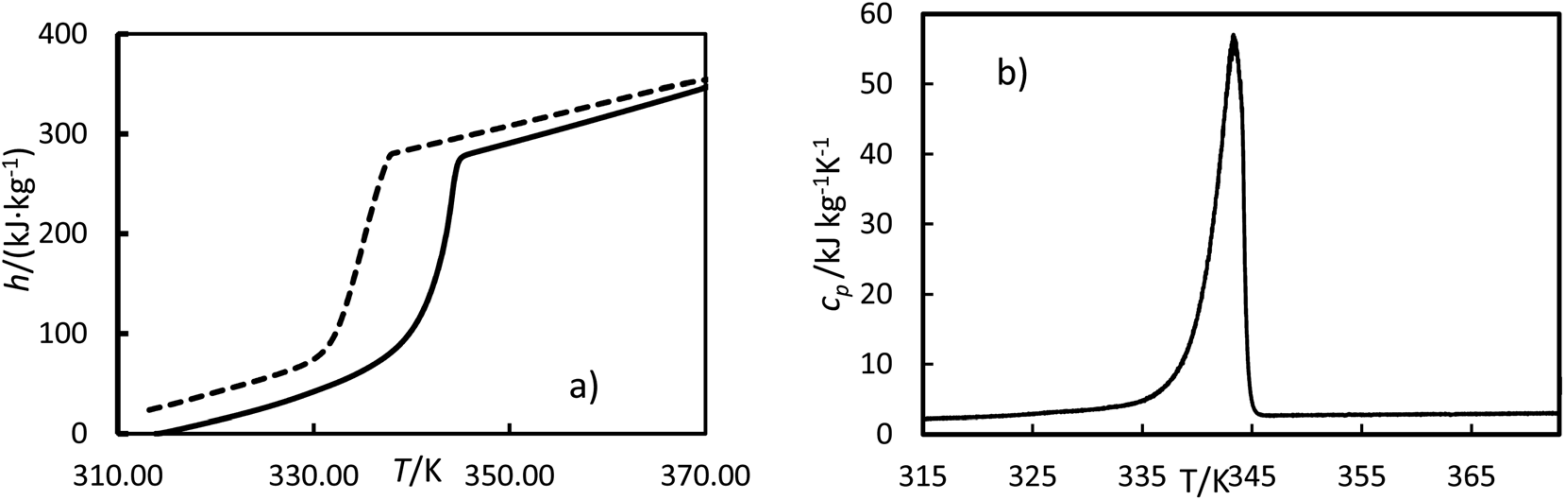
Experimental enthalpies, *h* (a) and heat capacity, *c*_p_ (b) *versus* temperature, *T*, for OM65 at 0.5 K min^−1^. The solid line is the heating cycle, and the dashed line is the cooling cycle.

The enthalpy curve is calculated by integrating the heat flow signal following eqn (2). Then, the heat capacity at constant pressure can be obtained following eqn (3).2
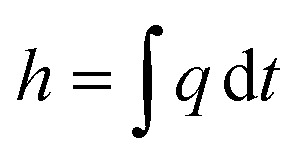
3
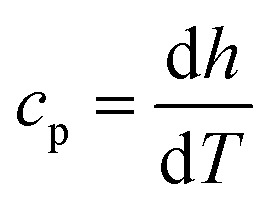
Here, *h* is the enthalpy, *q* is the specific heat flow, *t* is the time, *c*_p_, is the specific heat capacity, and *T* is the temperature. The enthalpy was calculated by using the trapezoid rule shown in eqn (4) and the *c*_p_ with eqn (5) with a program developed in MATLAB4
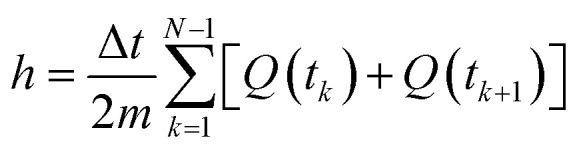
5
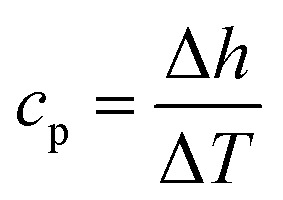
where *t* is time, *Q* is the heat flow at a particular time, and *m* is the mass. Examples of curves of enthalpy and heat capacity *versus* temperatures calculated are plotted in Fig. 4.

**Fig. 5:**
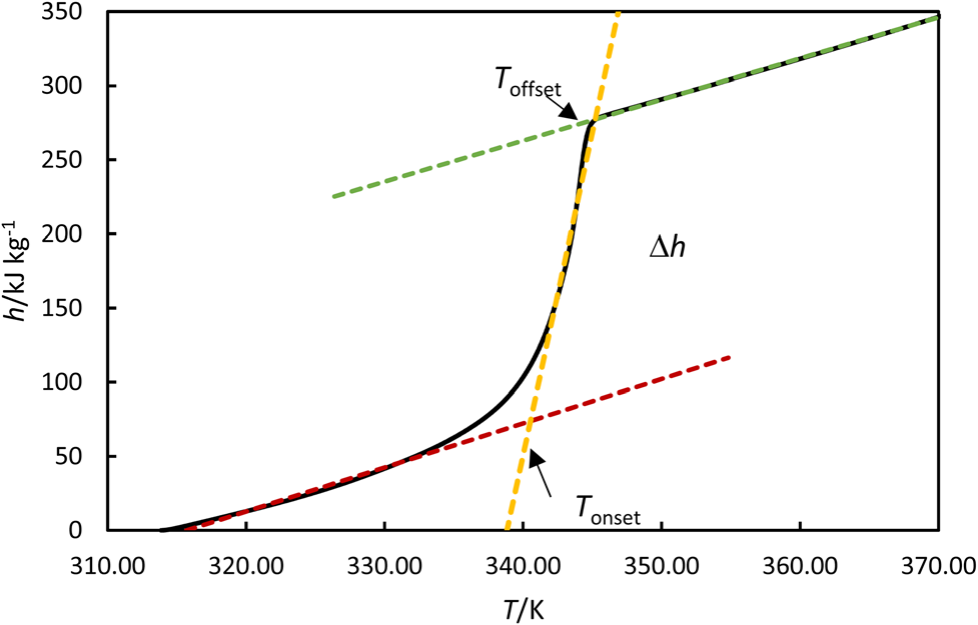
Experimental enthalpies *versus* temperature for OM65 at 0.5 K min^−1^. An example of determining the onset and offset temperatures, *T*_onset_ and *T*_offset_ and phase transition enthalpy, Δ*h*.

The melting enthalpy was calculated by fitting the data to three lines corresponding to the liquid, transition, and solid phases, as shown by the green, blue, and red lines for the heating cycle in Fig. 5. The intersection points between the liquid and transition lines and between the solid and transition lines represent the onset and offset temperatures, respectively. The enthalpy was determined from the differences between the enthalpies at these offset and onset temperatures. The solidification enthalpy was calculated using the same procedure for the cooling cycle. The melting and solidification temperatures were identified as the onset temperatures for the heating and cooling cycles, respectively. This procedure has been used in the literature to calculate melting and solidification enthalpies and melting and solidification temperatures for PCMs.^22^

#### 2.5.2 Measurement of the phase transition temperatures and enthalpies

Initial hf-DSC measurements are required to determine the lowest heating and cooling rate that allows thermal equilibrium^23–25^ to obtain accurate phase transition temperatures and enthalpies measurements as it is described on ESI.† The heating and cooling rates of 0.5 K min^−1^ was selected to reach the thermal equilibrium. Escribà *et al.* also chose 0.5 K min^−1^ heating rate to determine the phase change enthalpies for several ionic liquids.^13^

A SENSYS EVO TG-DSC instrument (Setaram, KEP Technologies Inc) was used to determine the phase transition temperatures and enthalpies in pure argon (Ar), 60 mL min^−1^ at 20 psi for each substance. This work used aluminium crucibles with a 30 μL volume and regular pressed lids. A balance (Mettler MX5, METTLER TOLEDO) with an accuracy of 0.01 mg was used to weigh the sample and empty crucibles. The DSC cycles were described as follows. About 20 mg of PCMs were placed into the sample crucible and held isothermally for 300 seconds at ambient temperature. Next, the furnace temperature was increased to 313 K at a heating rate of 10 K min^−1^ and held for 300 seconds. Then, a slow ramp was applied (0.5 K min^−1^) up to 373 K, then lowered to 313 K at 0.5 K min^−1^; this cycle was repeated three times. In the final cycle, the sample was held isothermally at 313 K for 300 seconds, ramped at 1 K min^−1^ to 373 K, and cooled to ambient conditions at 1 K min^−1^. The melting and solidification enthalpies, as well as the melting and solidification temperatures, were calculated following the procedure in Section 2.5.1 for both heating and cooling cycles, respectively. Each cycle was repeated four times, and the average values for each sample over these four cycles are reported. According to the DSC manufacturer, the temperature accuracy is ±0.1 K, heat flow resolution is 0.035 μW, and calorimetric precision is 0.1%. A blank subtraction was carried out using identical experimental conditions on an empty crucible to correct the effects of the changes in gas viscosity, density and buoyancy.

### 2.6 Thermal conductivity

The thermal conductivity of the solid and liquid samples was measured following the Transient Hot Bridge (THB) method using the THB-100 equipment from Linseis. A THB6K (42 × 22 mm) Kapton sensor and the same sensor with a metal frame (THB6K/MFR) were used to measure the solid samples, and the liquid samples, respectively. The thermal conductivity range was 0.02 to 5 W m^−1^ K^−1^ for a temperature range from −173 to 473 K.

#### 2.6.1 Solid phase measurements

**Fig. 6:**
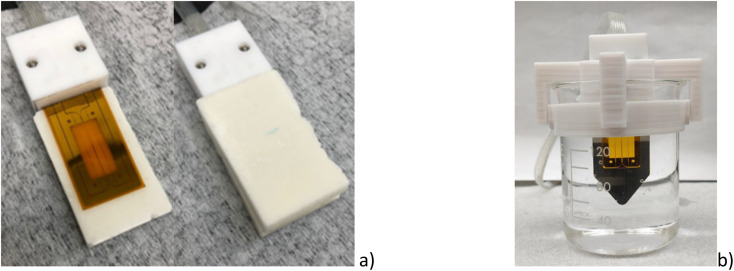
Thermal conductivity sensors for (a) the solid sample and (b) liquid sample including the support to hold the sensor.

The preparation of solid samples is reported on the ESI.† The sensor was placed between the two samples, as shown in Fig. 6a and a weight of 3 kg was placed on the top to ensure good thermal contact. At least three measurements consisting of five measuring points were conducted for each sample pair at room temperature. The uncertainty of the temperature was ±0.5 K. The heating current was adjusted to 60 mA, the measurement time to 30 s, and a delay time of 10 times the measuring time to allow the sample to cool down between measurements.

#### 2.6.2 Liquid phase measurements

The solid material was deposited on a 25 mL beaker and melted using a hot plate at about *T*_m_ + 5 K. Then, the beaker was immersed in a CC Immersion Circulator water bath from Huber. The temperature was controlled with an uncertainty of ±0.5 K. A 3D-printed support was designed in acrylonitrile butadiene styrene (ABS) to hold the sensor as shown in Fig. 6b. The thermal conductivity of the liquid samples was measured three times at different temperatures, from (343 to 358) K for each material. Each sample measurement consisted of five measuring points. The measuring time was 30 s at a measuring current of 40 mA. A pause of 60 s was made between the individual measurements to achieve constant temperature conditions.

## 3 Results

### 3.1 Viscosity

**Fig. 7:**
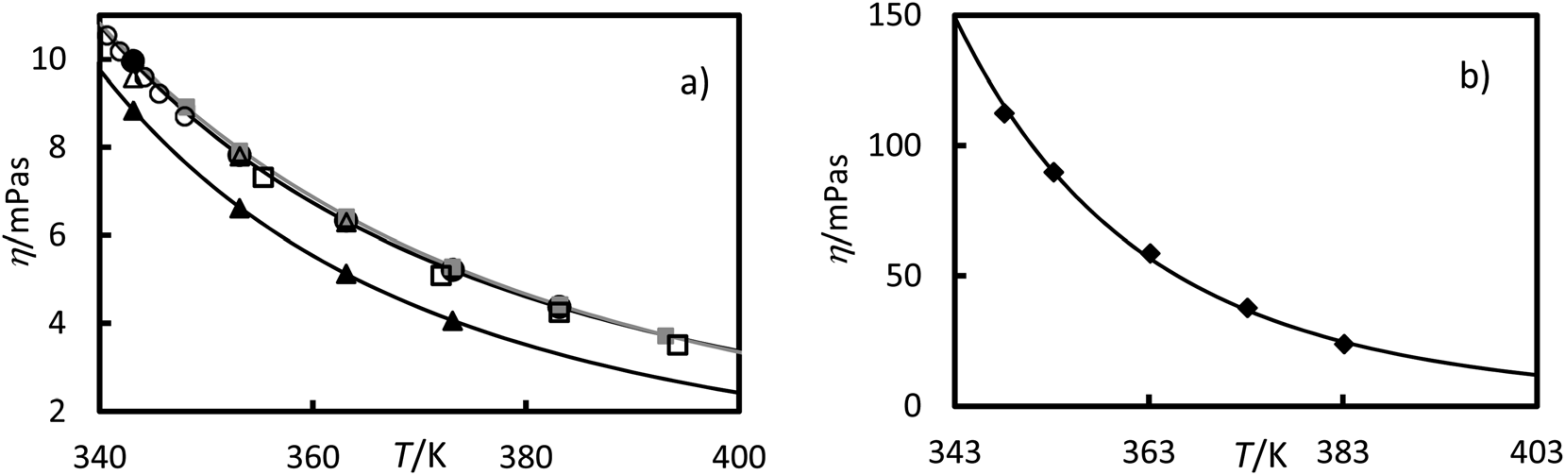
Experimental viscosities *versus* the temperature at 0.1 MPa for (a) ●, OM65, ▲, RTH64, and ■, stearic acid in this work and, stearic acid reported by □, Noureddini *et al.*;^26^ ○, G. Berchiesi *et al.*^27^ and △, Fernandez-Martin and Montes^28^ (b) ♦, [N_4444_^+^][Cl^−^]. The line represents the fitting of eqn (6) with the parameters of Table 3.

Viscosity measurements of the RT64HC, OM65, stearic acid and [N_4444_^+^][Cl^−^] were performed at temperatures ranging from 343 to 393.15 K at 0.1 MPa and are given in Table 2 and Fig. 7. As shown in Fig. 7, the viscosities of all studied materials decreased with increasing temperature, as expected. However, the viscosity reduction is more pronounced for the [N_4444_^+^][Cl^−^] than the other materials. It was found that [N_4444_^+^][Cl^−^] exhibited the highest viscosities, similar to other ionic liquids with the same cation, ammonium.^1^ Comparing the PCMs, the viscosities of stearic acid and OM65, were higher than the paraffin, RT64HC. Stearic acid and OM65 show similar viscosities values since both are fatty acids. Fig. 7a and Table 2 shows that there is a good agreement between the stearic acid viscosity measured in this study and the values reported in the literature. According to our knowledge, viscosity data for the other materials has never been published.

**Table 2:** Experimental viscosities (*η*) for the RT64H, OM65, stearic acid and [N_4444_^+^][Cl^−^] at temperatures, *T*. *u*(*η*) the viscosity uncertainty^a^

Substance		*T* (K)	*η* (mPa s)	*u*(*η*) (mPa s)
OM65	This work	343	9.96	0.19
		353	7.82	0.18
		363	6.33	0.17
		373	5.21	0.17
		383	4.37	0.16
RT64HC	This work	343	8.84	0.18
		353	6.61	0.17
		363	5.13	0.17
		373	4.06	0.17
Stearic acid	This work	348	8.92	0.18
		353	7.92	0.18
		363	6.42	0.17
		373	5.28	0.17
		383	4.42	0.16
		393	3.71	0.16
	Noureddini *et al.*^26^	355.4	7.31	
		372.1	5.08	
		383.2	4.24	
		394.3	3.50	
	G. Berchiesi *et al.*^27^	340.7	10.54	
		341.9	10.17	
		344.2	9.59	
		345.6	9.22	
		348.0	8.70	
	Fernandez-Martin and Montes^28^	343.15	9.583	
		353.15	7.794	
		363.15	6.294	
[N_4444_^+^][Cl^−^]	This work	348	112.5	3.3
		353	89.7	3.1
		363	58.7	2.7
		373	37.7	2.6
		383	23.7	2.5

a Standard uncertainties in temperatures *u*(*T*) = 1 K.

The viscosity data were fitted isothermally to the equation based on Vogel–Fulcher–Tammann (VFT).^29^6
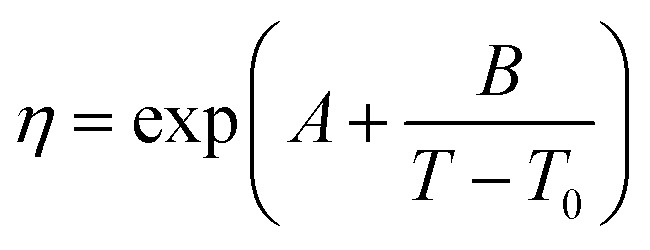
where *η* is the viscosity in mPa s, *T* is the temperature in Kelvin, and *A*, *B*, and *T*_0_ are fitting parameters. The Levenberg–Marquardt algorithm was used to minimise the sum of the squared differences between the experimental and calculated viscosities. The fitting parameters and absolute average deviation (AAD) and the absolute average relative deviation (AARD) are given in Table 3. These average deviations are similar to the experimental uncertainty, indicating that the equation can accurately represent the data. The highest average deviation and uncertainties were found for the ionic liquids, [N_4444_^+^][Cl^−^], this could be due to the absorption of small amounts of water.

**Table 3:** Parameters *A*, *B*, and *T*_0_ of eqn (6) for different substances. The absolute average deviation AAD, and absolute average relative deviation AARD of the correlation, eqn (6)

Substance	*A* (mPa s)	*B* (K)	*T*_0_ (K)	AAD (mPa s)	AARD (%)
OM65	−2.082	758.0	170.0	0.006	0.09
RTH64	−2.960	863.3	175.2	0.013	0.17
Stearic acid	−3.282	1298.0	110.8	0.008	0.12
[N_4444_^+^][Cl^−^]	−8.708	3632.0	78.2	0.700	0.60

### 3.2 Density

The measured solid densities of the RT64HC, OM65 and stearic acid are given in Table 4 together with the values reported. It can be noted that the density of the paraffin is the lowest and the density of ionic liquid is the highest. As shown in Table 4, the experimental densities reasonably agree with the data reported.

**Table 4:** Experimental densities (*ρ*) of the several substances at room temperature and atmospheric pressure in this work. *ρ*_lit_ is the density found in the literature

Substance	*T* (K)	*ρ* (kg m^−3^)	*u*(*ρ*) (kg m^−3^)	*ρ*_lit_ (kg m^−3^)
OM65	294.2	921	2	924 (303 K)^a^
RT64HC	293.2	891	3	880 (293 K)^a^
Stearic acid	287.1	938	3	941 (293 K)^b^
[N_4444_^+^][Cl^−^]				1050 (293 K)^a^

a Data reported from manufacture.

b Data reported by ref. 30.

### 3.3 Thermogravimetric analysis

**Fig. 8:**
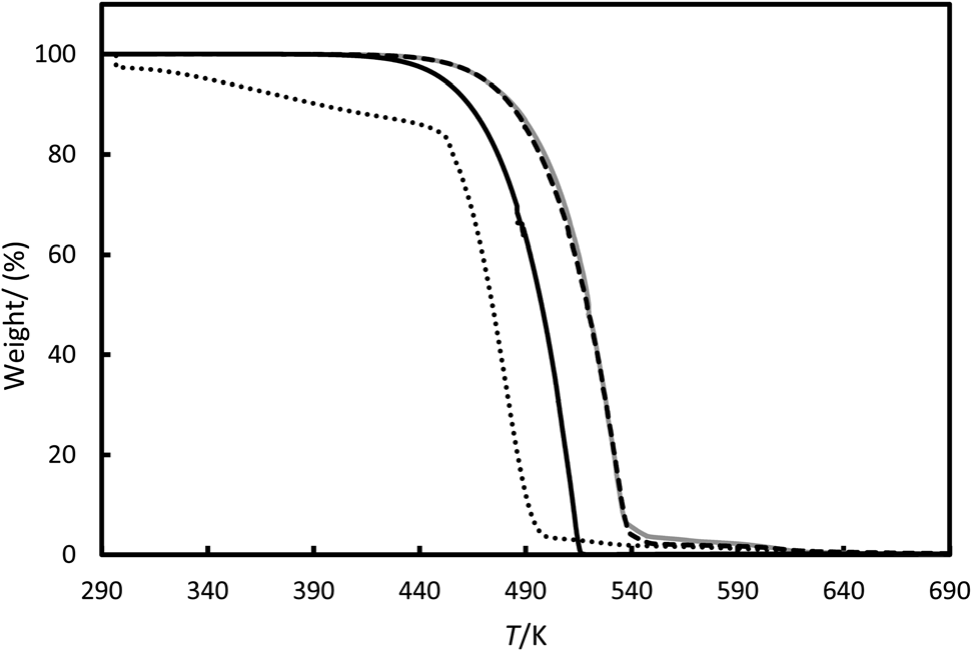
Thermogravimetric analysis (TGA) for the (---) OM65, (—) RT64H, (grey line) stearic acid and (⋯) [N_4444_^+^][Cl^−^].

Fig. 8 shows the mass loss *versus* temperature for the RT64HC, OM65, stearic acid and [N_4444_^+^][Cl^−^] at heating rate 1 K min^−1^. The TGA showed relatively high mass losses from about 490 K to 560 K for RT64HC, OM65 and stearic acid due to the material decomposition by the breakup of the O–C and C–C bonds. Two characteristic mass losses were identified for the [N_4444_^+^][Cl^−^], first between 301 to 383 K and second between 450 to 500 K. The first was related to the desorption of water sorbed by the ionic liquid, whereas the second is the most considerable mass loss due to the degradation of the ionic liquid by the breakup of the N–C and the C–C bonds. For all the studied materials, the residue mass after about 640 K was lower than 0.01%, indicating the total degradation.

The amount of water sorbed on the materials was determined by the change in mass below 383 K and it is shown in Table 5. The water content for the PCMs was lower than 1%. The average water content of the ionic liquids was up to 10.0% with a standard deviation of 0.6%, slightly higher than the manufacturer's specifications. This indicates that the ionic liquids likely absorbed some water during sample manipulation, consistent with the well-known hygroscopic nature of these substances.^31^ The decomposition temperatures (*T*_d_) are higher for the PCMs than for [N_4444_^+^][Cl^−^] indicating better thermal stability. Other authors in the literature reported similar *T*_d_ for tetrabutylammonium cations-based ionic liquids.^11^ The TGA shows that all the studied PCMs are thermal stable until about 450 K, so they can efficiently work in domestic applications.

**Table 5:** Water content in mass per cent (%) and decomposition temperature (*T*_d_) for different substances studied from the TGA. *u*(*T*_d_) denotes the uncertainty in the *T*_d_

Substance	Water content (%)	*T*_d_ (K)	*u*(*T*_d_)
RT64HC	0.05	475.4	0.4
OM65	0.06	499.6	0.2
Stearic acid	0.03	492.6	0.4
[N_4444_^+^][Cl^−^]	10.0	439.7	0.5

### 3.4 Phase change temperatures and enthalpies

**Fig. 9:**
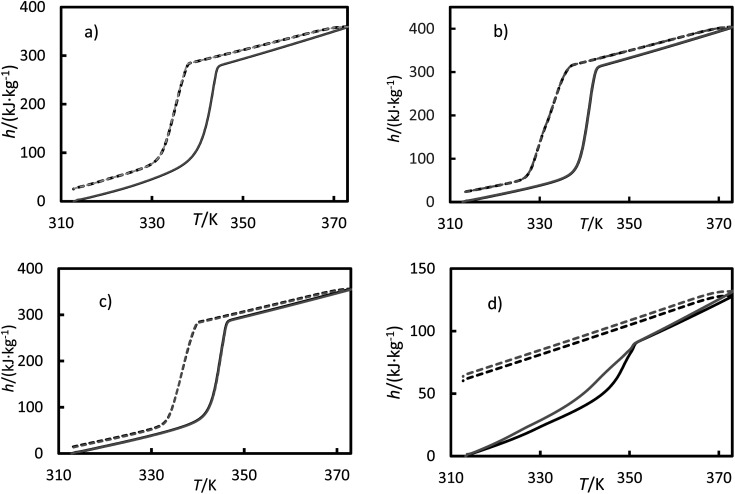
Enthalpies *versus* temperatures for (a) the RT64HC, (b) OM65, (c) stearic acid and (d) [N_4444_^+^][Cl^−^] from a DSC measurement. Solid curves represent the heating ramp, and the dashed line represents the cooling ramp. Black represents the second cycle, and gray represents the third cycle.

The measured enthalpy, phase change temperature and uncertainties are given in Tables 6 and 7. Fig. 9 shows the enthalpies for the (a) RT64H, (b) OM65, (c) stearic acid and (d) [N_4444_^+^][Cl^−^] during the heating and cooling for the second and third cycles, for clarity. The melting and solidification enthalpies for PCMs were significantly higher than for the ionic liquid, as seen in Fig. 9. In fact, the change in enthalpy in the ionic liquid during the solidification process was not observed in the results and the material remains in liquid phase. It can also be seen a hysteresis between the heating and cooling cycle caused by subcooling. A metastable state is formed during the cooling so that the nucleation temperature would be lower than the melting temperature.

**Table 6:** Melting temperature, *T*_m_ and melting enthalpy, Δ*h*_m_ for OM65, RTH64, stearic acid and [N_4444_^+^][Cl^−^] obtained from DSC data and the literature. *u* denote the uncertainty for each property

Substance	*T*_m_ (K)	*u*(*T*_m_) (K)	Δ*h*_m_ (kJ kg^−1^)	*u*(Δ*h*_m_)	Literature data		
					*T*_m_ (K)	Δ*h*_m_ (kJ kg^−1^)	Reference
OM65	341.5	0.2	203	2	341	183	a
RT64HC	339.6	0.2	252	4	337	250	a
Stearic acid	343.4	0.2	214	5	338.4	210	32
					342.2	199	33
					342.0	214.7	34
					343.52	212.2	35
[N_4444_^+^][Cl^−^]	346.4	0.2	29.2	1	314.1	20.5	36
					348.1		37
					347.15		38
					350.15		39

a Data obtained by manufacture specifications.

**Table 7:** Solidification temperature, *T*_s_ and solidification enthalpy, Δ*h*_s_ for OM65, RTH64 and stearic acid obtained from DSC data and the literature. *u* denote the uncertainty for each property

Substance	*T*_s_ (K)	*u*(*T*_s_) (K)	Δ*h*_s_ (kJ kg^−1^)	*u*(Δ*h*_s_)	Literature data		
					*T*_s_ (K)	Δ*h*_s_ (kJ kg^−1^)	Reference
OM65	337.8	0.4	180	4	338		a
RTH64	337.5	0.7	222	5	337		a
Stearic acid	338.8	0.2	170	7	342.1	212.1	40
					341.2	211.46	35

a Data obtained by manufacturer specifications.

The melting temperatures ranged from 340 to 346 K for all the materials, and the solidification temperatures were about 338 K for the PCMs studied in this work. The solidification temperature could not be obtained from DSC data for the ionic liquids. It can be found that the temperature differences between the melting and solidification temperatures are in about 1–5 K range. This value range is very low, indicating the low subcooling tendency of these PCMs. As seen from the Table 6, the temperatures agree with the data provided in the literature, except for data reported by Coker *et al.* for the ionic liquid.^36^ The melting enthalpies for studied PCMs ranged from 200 to 250 kJ kg^−1^, obtaining lower values for the fatty acids. Also, the solidification enthalpies (Table 7) are lower than the melting enthalpy by about 20 kJ kg^−1^. The enthalpies reported in Tables 6 and 7 are in good agreement with the literature. The small difference between our data and the literature data could be due to variations in techniques, experimental uncertainties, and other factors. The ionic liquid showed the lowest melting enthalpy, 29.2 kJ kg^−1^. This value is in agreement with the value measured by Coker *et al.*^36^ Other ionic liquids based on ammonium cation, such as tetrabutylammonium thiocyanate^41^ and tetrabutylammonium perchlorate^42^ shown as well low phase transition enthalpies.

### 3.5 Specific heat capacity

**Fig. 10:**
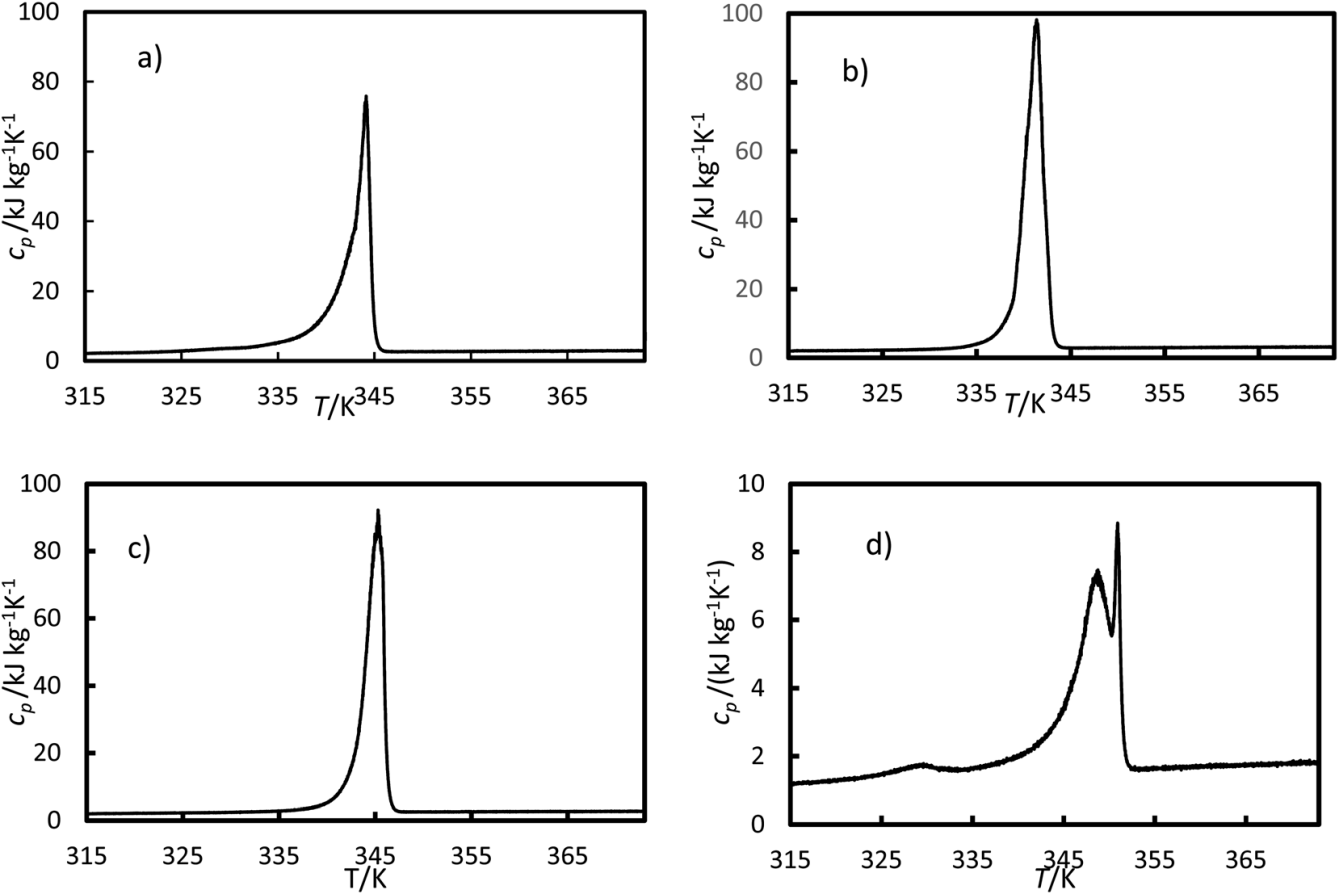
Specific heat capacity *versus* temperatures for (a) the RT64HC, (b) OM65, (c) stearic acid and (d) [N_4444_^+^][Cl^−^] were obtained using eqn (5) and data from the DSC measurement.

The specific heat capacity was calculated using eqn (5) for the heating ramp and is presented in Fig. 10. For the ionic liquids, two peaks were registered during the transition (mushy zone).

In this work, the solid heat capacities for the four cycles *versus* temperature were fitted to a polynomial of second order, eqn (7).

7 *c*_p_ = *A* + *BT* + *CT*^2^

The parameters for the solid heat capacity for all studied materials are listed in Table 8. The standard deviation between the experimental and calculated values is also reported in Table 8, ranging from 0.04 to 0.2 kJ kg^−1^ K^−1^. As can be seen, the calculated average solid heat capacity agrees with the data reported by manufacturers and literature. Since the specific heat capacity for the liquid phase was almost constant in the temperature range studied here, only the average value and the standard deviation were reported in Table 9. In general, the heat capacities for the studied PCMs were higher than the ones for the ionic liquid. Furthermore, the heat capacities for the solid phase were consistently lower than for the liquid phase. Bhatt and Gohil reported similar heat capacities for another ionic liquid with the same cation, tetrabutylammonium.^11^

**Table 8:** Parameters of eqn (7) for the heat capacity of the solid phase as a function of the temperature of several substances. *T* range denotes the temperature range where the equation can be applied, SE is the standard error of each parameter and *s* is the standard deviation between the experimental and calculated data. *c*_p_ is the average experimental specific heat capacity, and *c*^li^_p_ is found in the literature

Substance	*A* (kJ kg^−1^ K^−1^)	SE (kJ kg^−1^ K^−1^)	*B* (kJ kg^−1^ K^−2^)	SE (kJ kg^−1^ K^−2^)	*C* × 10^3^ (kJ kg^−1^ K^−3^)	SE × 10^3^ (kJ kg^−1^ K^−3^)	*s* (kJ kg^−1^ K^−1^)	*T* range (K)	*c*_p_ (kJ kg^−1^ K^−1^)	*c* ^li^_p_ (kJ kg^−1^ K^−1^)	
									Average	Literature	
OM65	425	10	−2.71	0.6	4.34	0.10	0.08	315–330	2.69	2.83^a^	303.15
RT64HC	184	8	−1.16	0.5	1.85	0.08	0.05	315–330	2.24	2.00^a^	
Stearic acid	213	4	−1.33	0.03	2.10	0.04	0.04	315–333	2.32	2.83 (ref. 40)	313.00
[N_4444_^+^][Cl^−^]	−8.42	0.27	0.031	8.2 × 10^−4^	0		0.20	315–336	1.68		

a Data obtained by manufacturer specifications.

**Table 9:** The average experimental liquid specific heat capacity *c*_*p*_. *s* denotes the standard deviation between the experimental and calculated data. *T* range indicates the temperature range where the average value was calculated. *c*^lit^_p_ heat capacity found in the literature

Substance	*c*_p_ (kJ kg^−1^ K^−1^)	*s* (kJ kg^−1^ K^−1^)	*T* range (K)	*c* ^lit^_p_ (kJ kg^−1^ K^−1^), literature
OM65	2.80	0.09	350–372	2.38^a^
RT64HC	3.00	0.10	350–372	2.00^a^
Stearic acid	2.59	0.09	350–372	2.38 (353 K)^40^
[N_4444_^+^][Cl^−^]	1.80	0.09	355–370	

a Data obtained by manufacturer specifications.

### 3.6 Thermal conductivity

The thermal conductivity for the solid phase of RT64HC, OM65, stearic acid, and [N_4444_^+^][Cl^−^] were determined at room temperature and are given in Table 10. The PCMs presented similar conductivity within the range (0.240 to 0.270) W m^−1^ K^−1^. The lowest solid thermal conductivity value was found for [N_4444_^+^][Cl^−^]. Similar thermal conductivity was found for other ionic liquids with the same cation, ammonium, in the literature.^1^ The thermal conductivities values obtained for the RT64HC and OM65 are slightly higher than the data reported by manufacturers. However, the thermal conductivity measured for stearic acid in this work is lower than the data reported in the literature. It is important highly that there is a significant difference in the literature.

**Table 10:** In this work, experimental thermal conductivity (*λ*) of solid phase for several substances at room temperature and atmospheric pressure. Data reported in the literature or by the manufacturer

Substance	*T* (K)	*λ* (W m^−1^ K^−1^)	*u*(*λ*) (W m^−1^ K^−1^)	Literature data		
				*λ* (W m^−1^ K^−1^)	*T* (K)	Reference
OM65	293.6	0.242	0.007	0.19^a^	303	
RT64HC	291.9	0.281	0.009	0.200^a^		
	294.2	0.274	0.009	0.200^a^		
Stearic acid	293.3	0.270	0.008	0.410	298	43
				0.290		44
				0.300		45
[N_4444_^+^][Cl^−^]	290.1	0.164	0.007			
	289.0	0.175	0.007			

a Data reported from manufacture.

**Fig. 11:**
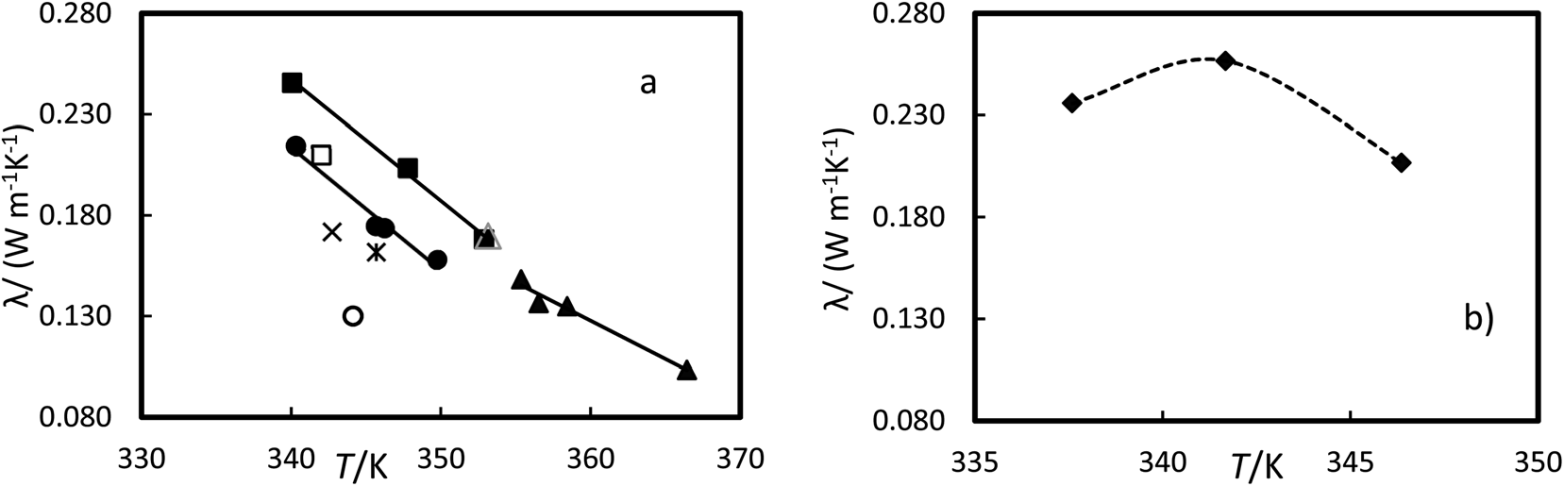
Experimental thermal conductivity *versus* the temperature at 0.1 MPa for (a) ●, OM65, ▲, RTH64, and ■, stearic acid in this work and stearic acid reported by △ Mukhamedzyanov,^46^ □, Bayram *et al.*,^47^ ✕, Hopfe^48^ and ✻ Kern and Nostrand^49^ and OM65 reported by ○, manufacture. (b) ♦, [N_4444_^+^][Cl^−^]. The line represents the fitting of the equation with parameters of Table 12 and the dash line as guide to the eyes.

The thermal conductivity for the liquid phase of RT64HC, OM65, stearic acid and [N_4444_^+^][Cl^−^] were determined as a function of the temperature at atmospheric pressure and are given in Table 11 and Fig. 11. The thermal conductivity for the liquid phase could be measured at temperatures lower than the melting temperatures and higher than the solidification temperature due to the subcooling effect since the material was melting at about 358 K and cool down to the measured temperature (see Fig. 9). The range of liquid thermal conductivities for the PCMs was from (0.24 to 0.10) W m^−1^ K^−1^. The highest values of conductivity, about 0.26 W m^−1^ K^−1^ were found for the ionic liquid. As it is expected, the liquid thermal conductivities decreased with the temperature. Fig. 11 represents the data from the manufacturer and literature for the OM65 and stearic acid, respectively. The measured liquid thermal conductivity for OM65 were higher than the ones reported by the manufacturer. And for stearic acid they agree with data reported by Mukhamedzyanov,^46^ which were slightly higher than other data reported from the literature. In the studied conditions, the liquid phase conductivity for the ionic liquid at first increased with temperature until a maximum was reached, after which the values decreased (Fig. 11b). This behaviour could be due to the metastable phase of the ionic liquid at 337.6 K.

**Table 11:** Experimental thermal conductivity (*λ*) of liquid phase for the several substances as a function of temperature and atmospheric pressure in this work

Substance	*T* (K)	*λ* (W m^−1^ K^−1^)	*u*(*λ*)
OM65	340.3	0.214	0.026
	345.6	0.175	0.013
	346.2	0.174	0.012
	349.8	0.158	0.008
RT64HC	355.4	0.148	0.005
	356.5	0.137	0.007
	358.4	0.135	0.005
	366.4	0.103	0.008
Stearic acid	340.1	0.246	0.010
	347.8	0.204	0.010
	352.9	0.168	0.007
[N_4444_^+^][Cl^−^]	337.6	0.236	0.020
	341.7	0.257	0.015
	346.4	0.207	0.021

The liquid thermal conductivity for the PCMs studied decreases linearly with the temperature. It was fitted to a linear correlation of the form:

8 *λ* = *CT* + *D*

where *C* and *D* are parameters of the equation and *T*, is temperature in Kelvin. *C* and *D*, together with the AAD and AARD are given in Table 12. These AADs are less than the experimental uncertainty, indicating that the equation can accurately represent the data. The [N_4444_^+^][Cl^−^] thermal conductivity have the highest uncertainties. This could be the absorption of small quantities of water by ionic liquid.

**Table 12:** Parameters *C* and *D* of eqn (8) for different substances. The absolute average deviation AAD, and absolute average relative deviation AARD of the correlation

	*C* × 10^3^ (W m^−1^ K^−2^)	*D* (W m^−1^ K^−1^)	AAD (W m^−1^ K^−1^)	AARD (%)
OM65	−6.096	2.286	0.003	1.8
RT64H	−3.787	1.491	0.002	1.6
SA	−5.999	2.287	0.002	1.2

## 4 Discussion

This section compares the properties of the ionic liquid with the PCMs investigated and with common organic-based HTFs, such as Therminol VP-1 use in solar collector applications. Table 13 gives the properties of the compound mentioned.

**Table 13:** Thermophysical properties of Therminol VP-1 reported by manufacturer and OM65, RT64HC, stearic acid and [N_4444_^+^][Cl^−^] measured in this paper. *ρ* denotes the liquid density; *η*, viscosity; *T*_m_, melting temperature; *h*_m_ melting enthalpy; *c*_p_ specific heat capacity and *λ*, thermal conductivity

		OM65	RT64HC	Stearic acid	[N_4444_^+^][Cl^−^]	Therminol VP-1^a^
Applicability temperature range liquid (K)		341.5–499.6	339.6–475.4	343.4–492.6	346.4–439.7	285 to 673
*ρ* (kg m^−3^)		921 (294 K)	891 (293 K)	938 (287 K)	1050 (293 K)^a^	1060 (298 K)
*η* (mPa s)	At 353 K	7.82	6.61	7.92	89.7	1.28
	At 373 K	5.21	4.06	5.28	37.7	0.99
*T*_m_ (K)		341.5	339.6	343.4	346.4	285
*h*_m_ (kJ kg^−1^)		203	252	214	29.2	97.3
*c*_p_ at 353 K (kJ kg^−1^ K^−1^)		2.80	3.00	2.59	1.80	1.719
*λ* (W m^−1^ K^−1^)	At ambient temperature	0.281 (293 K)	0.274 (294 K)	0.270 (293 K)	0.164 (290 K)	
	At 345 K	0.195^b^	0.192^b^	0.229^b^	0.256^c^	0.131

a Data reported by manufacturer.

b Data calculated using eqn (8) and the parameters Table 12.

c Interpolates from the data in Table 13.

The viscosity of the ILs is significantly greater than the PCMs investigated and HTFs, see Table 13. This would increase the operational costs of the pump for a heat exchange system or decrease the natural convention of the melting or solidification process of the PCMs. However, the viscosity of ILs decreases substantially with temperature Fig. 7 by up to 80% when the temperature increases by 40 K. This could increase the effectiveness of heat and mass transport in the process for application at high temperatures. From Table 13, the density for IL is similar to Therminol VP-1 and slightly higher than the PCMs. Regarding thermal stability, the ILs studied showed high stability from ambient conditions up to about 440 K, similar to organic PCMs or organic HTFs, which is suitable for domestic application.

The phase change enthalpy indicates the amount of heat a material can absorb or release during its transformation. Materials with phase change enthalpies of more than 100 kJ kg^−1^ are regarded as suitable candidates for use as PCM_s_, and this is the average thermal energy storage capacity of commercial PCMs, according to the literature. The [N_4444_^+^][Cl^−^] presents a phase change temperature of 346.4 K similar to studied organic PCMs. However, the phase change enthalpy is very small, about 30 kJ kg ^−1^, compared to the PCMs, about 200 kJ kg ^−1^. The specific heat capacity (*c*_p_) is also crucial for the heat transfer processes and determining sensible heat storage capacity. The average *c*_p_ of [N_4444_^+^][Cl^−^] were much lower than the studied PCMs and similar to HTFs, see Table 13.

The thermal conductivity is essential for the heat transfer process. The thermal conductivity value for the solid phase was lower for the IL than for the PCMs. However, the thermal conductivity values for the liquid phase were the highest, see Table 13. They are sufficient for space and water heating.

[N_4444_^+^][Cl^−^] exhibits heat capacity, thermal conductivity, and thermal stability comparable to commonly used heat transfer fluids (HTFs), making it a viable alternative in solar collectors for domestic water heating, for example, with the added advantage of low vapor pressure, which is beneficial for high-temperature applications. Additionally, at temperatures above 350 K, [N_4444_^+^][Cl^−^] can store sensible heat without undergoing a phase change, minimizing volume changes, eliminating the complexity of handling two phases, and reducing the subcooling effect. However, its low phase change enthalpy of about 30 kJ kg^−1^, significantly lower than that of organic PCMs, makes it unsuitable for latent heat storage devices. In summary, [N_4444_^+^][Cl^−^] exhibits several strengths that make it a viable heat transfer fluid for specific applications, especially where low vapor pressure, high thermal conductivity and thermal stability are critical. However, its lower energy storage capacity compared to organic PCMs presents a significant weakness. There are opportunities in domestic water heating and as an alternative to organic HTFs, but market acceptance and competition with established materials pose threats to its widespread adoption.

One of the limitations of organic PCMs is their low thermal conductivity. The dispersion of highly conductive nanoparticles on the organic material is one of the techniques used to improve the thermal conductivity of PCMs.^5^ However, the nanoparticles tend to agglomerate and then they might precipitate.^50^ In this context, [N_4444_^+^][Cl^−^] can be potentially used as a surfactant to disperse nanoparticles on traditional PCMs, avoiding the aggregation and precipitation problems of nanoparticles, as it seems in the literature.^51^ Furthermore, the [N_4444_^+^][Cl^−^] can form semiclathrate hydrates and semiclathrate hydrates could be used as energy storage as has been the case with other ionic liquids-based on tetrabutylammonium anion.^52^ In addition, TBAC, as a hydrogen-bond acceptor, has been used in the preparation of deep eutectic solvents (DES)^53^ that have the potential to be used in thermal energy storage.^54^

## 5 Conclusions

The viscosity, density, thermal stability, phase change temperatures and enthalpies, specific heat capacity and thermal conductivity data have been measured at atmospheric pressure for tetrabutylammonium chloride and commonly used PCMs two fatty acids (OM65 and stearic acid) and one paraffin (RT64HC). The properties of tetrabutylammonium chloride are compared with widely used HTFs such as Therminol VP-1. The uncertainties are determined, and their values depend on the properties. These data agree well with existing literature or manufacture data. Two correlations were applied to fit the viscosity and thermal conductivity data as a function of temperature, and these adjusted the data within the reported uncertainty. [N_4444_^+^][Cl^−^] is not recommend for latent heat storage applications, since its phase change enthalpy is only about 30 kJ kg^−1^. On the other hand, [N_4444_^+^][Cl^−^] possesses properties, such as heat capacity, thermal conductivity, and thermal stability, comparable to those of commonly used heat transfer fluids, allowing for its effective application as a heat transfer fluid such as heat exchanger. The feasibility to use [N_4444_^+^][Cl^−^] as surfactant for nano-enhanced PCMs must be investigated in the future.

## Abbreviations

LHTES: Latent heat thermal energy storage
[N_4444_^+^][Cl^−^]: Tetrabutylammonium chloride
PCM: Phase change material
TES: Thermal energy storage

### Symbols

*c*_p_
: Specific heat (J kg^−1^ K^−1^)
*E*_a_
: Activation energy (J mol^−1^)
h
: Enthalpy (J kg^−1^ K^−1^)
M
: Molar mass
*m*_air_
: Weigh in air (kg)
*m*_E_
: Weigh in ethanol (kg)
q
: Specific heat flow (W)
R
: Gas constant (8.3144 J mol^−1^ K^−1^)
T
: Temperature (K)
*T*_m_
: Melting temperature (K)
t
: Time (s)

### Greek symbols

λ
: Thermal conductivity (W m^−1^ K^−1^)
x
: Mole fraction
ρ
: Density (kg m^−3^)
η
: Dynamic viscosity (mPa s)

### Subscripts

*i*
: Element number
m: Melting
s: Solid
l: Liquid

## Data availability

The authors state that the data supporting the findings of this study can be found within the article and/or its ESI.†

## Conflicts of interest

There are no conflicts to declare.

## Supplementary Material

RA-014-D4RA03782K-s001
