# Supplementary material for: Thermophysical properties of tetrabutylammonium chloride, paraffin and fatty acids for thermal energy applications
Source: RSC Adv. 2024 Aug 19;14(36):26246–58. doi: 10.1039/d4ra03782k (PMC11332589; doi:10.1039/d4ra03782k)
Supplement: RA-014-D4RA03782K-s001 [file RA-014-D4RA03782K-s001.pdf]

Supplementary Information (SI) for RSC Advances.
This journal is © The Royal Society of Chemistry 2024

Thermophysical Properties of Tetrabutylammonium chloride, paraffin and fatty acids for Thermal Energy Applications

Tomás Costa[a], Yolanda Sanchez-Vicente[a*] , Zili Yang[a], Lee A. Stevens[b], Fabio de S. Dias[c], Sol-Carolina Costa[a,c*]

[a] Department of Mechanical and Construction Engineering, Northumbria University, Wynne-Jones Centre, Newcastle upon Tyne NE1 8ST, United Kingdom

[b] University of Nottingham, Low Carbon Energy and Resources Technologies Group, Faculty of Engineering, Energy Technologies Building, Triumph Road, Nottingham, NG7 2TU, United Kingdom

[b] Federal University of Bahia (UFBA), Institute of Chemistry, Salvador, Brazil

[c] School of Computing, Engineering & the Built Environment, Edinburgh Napier University, EH10 5DT, UK

Supplementary Information

1.- Experimental Uncertainty

1.2- Viscosity

The viscometer was calibrated using a standard oil from Merck that covers viscosity from 4 to 1 mPa s^{-1} and HT-3000 calibration oil from Brookfield with viscosity from 9000 to 4500 mPa·s. The experimental mean values and estimated uncertainties are reported in this study.

Validation

Standard oil which is a mixture of odourless kerosene and white mineral oil from Merck. The oil is a general-purpose viscosity standard certified by ISO 17034 and UKAS ISO/IEC17025. The procedure was the same than the one described in the main manuscript. The experimental viscosity data of the Standard oil were obtained at temperatures ranging from (293 to 303) K at ambient pressure. Since the lowest viscosity limit for Brookfield viscometer is 3 mPa·s, so the viscosity measurements couldn't be conducted at higher temperatures. Table S1 compares our measured data with the standard reference data from Paragon Scientific. As can be seen from the Table S1, deviations were about 0.2 mPa·s. These values were similar to our uncertainty.

Table S1. The viscosity of the standard oil measured in this work, η_{exp} and the standard reference values. (η_{ex}-η_{st}) is the difference between the two values

T/K	η_{exp} (mPa·s)	$\eta_{standard}$ (mPa·s)	(η_{ex}-η_{st})/ (mPa·s)
293	4.010	3.733	0.28
298	3.566	3.283	0.28
303	3.137	2.960	0.18

Furthermore, the viscometer calibration was verified with HT-3000 fluid recommended by the Brookfield Thermosel System.

Uncertainty calculations

The uncertainty in the viscosity was caused by the direct measurement of the viscosity, which according to manufacturer specifications was approximately 1%. There are also stochastic uncertainties that influence the measurements, including sample preparation, spindle alignment, thermal stability, and vibration, among others. The random uncertainties were calculated using the standard uncertainty that is define as $u_R(\eta)=s/\sqrt{n}$, where s is the standard deviation and n is the number of repetitions. Then, temperature fluctuations also affect to uncertainty of viscosity. According with the guidelines described in the GUM, the viscosity uncertainties can be calculated with the following equation:

$$u(\eta)^2 = u(\eta)^2 + u_R(\eta)^2 + \left(\frac{d\eta}{dT}\right)^2 u(\eta)^2$$

1.3.-Density

The uncertainty for the density measurements was due to the equipment, the materials, presence of air bubbles, environmental conditions such as vibration, mass measurements, uncertainty of ethanol

and air densities and the temperature measurements. According with the guidelines described in the GUM, the density uncertainties can be calculated with the following equation:

$$u(\rho)^2 = u(\rho)^2 + \left(\frac{d\rho}{dm_E}\right)^2 u(m_E)^2 + \left(\frac{d\rho}{dm_A}\right)^2 u(m_A)^2 + \left(\frac{d\rho}{d\rho_E}\right)^2 u(\rho_E)^2 + \left(\frac{d\rho}{d\rho a}\right)^2 u(\rho_a)^2$$

Where $u(\rho) = s/\sqrt{n}$ is the standard uncertainty due to random error such as the vibrations, air bubble, the skill of the operator, temperature fluctuation and so on. m is the mass , T is the temperature, ρ_E and ρ_A are the density of ethanol and air respectively.

1.4 Thermogravimetric Analysis

Thermogravimetric Analysis (TGA) evaluate the thermal stability by determining the mass change of a material as a function of temperature under gas flow. A TA Instruments Q-550 was used, the samples were heated up to 800 K at 10 K min^{-1}, 5 K min^{-1} and 1 K min^{-1} under nitrogen flow to prevent oxidation during the measurement. Thermogravimetric graph (TGA) for each sample at three heating rates are presented in Figure S.1.

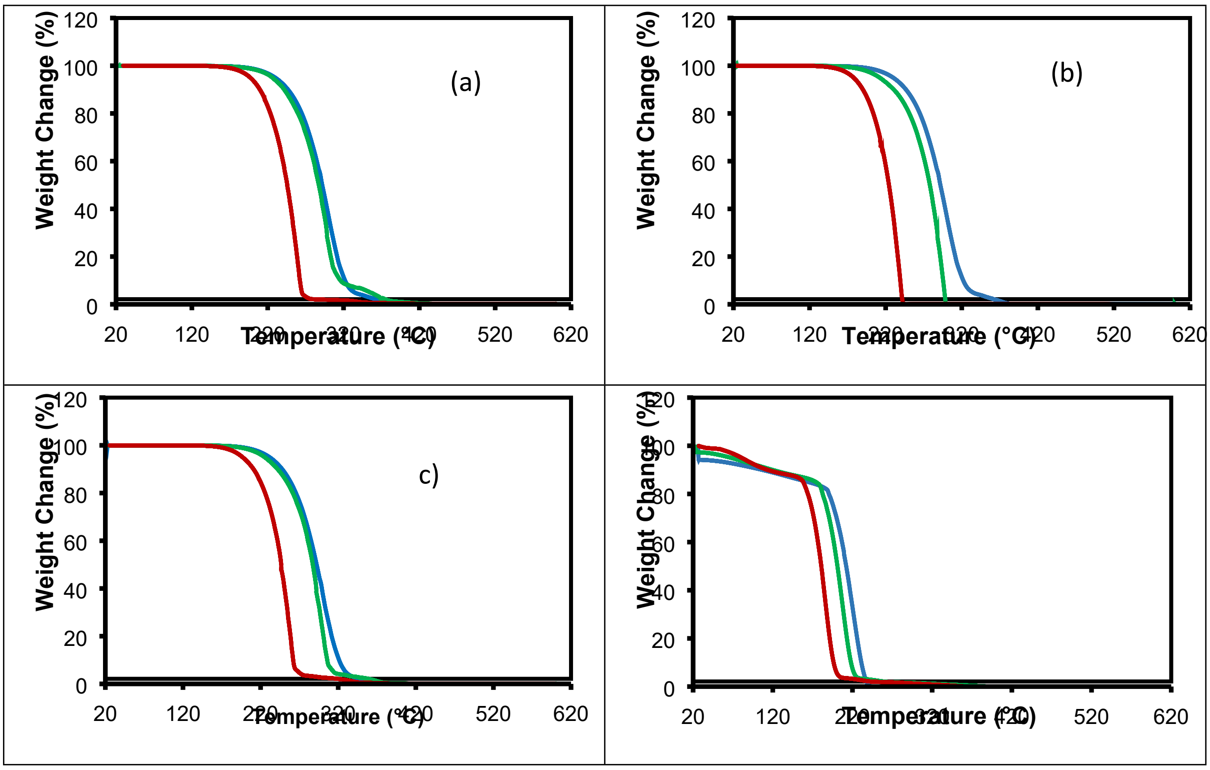

Figure S.1. Thermogravimetric Analysis (TGA) for the (a)OM65, (b)RT64H, (c) Stearic acid and (d) [N_{4444}^+][Cl^-] at 10 °C min^{-1} (blue); at 5 °C min^{-1}(green); and at 1 °C min^{-1}(red).

The uncertainty in the thermal decomposition temperatures was estimated to be about ±0.5 K and was calculated by the standard error that is defined by $u(T_d) = \frac{s}{\sqrt{n}}$; where *s* is the standard deviation, and n is the number of repetitions.

1.5 Phase transition temperatures and enthalpies

Selection of heating and cooling rates: Six cooling and heating rates were tested (10; 5; 2.5; 1.25; 0.5 and 0.2 K min^{-1}) starting at 10 K min^{-1} and reduced by a half in the follow cycle[27]. According to RAL-

GZ 896, the correct heating rate can be found by finding the temperature difference between the inflexion points of two close heating and cooling ramps from enthalpy versus temperature graphs. The difference between the inflexion temperatures for the two lowest heating rates of 0.5 and 0.2 K min^{-1}, were 0.6 and 0.5 K. Therefore,

The uncertainty in the melting and solidification temperatures resulted from the direct measurement of the temperature, which according to the manufacturer's specifications was 1%. There are also random uncertainties that impact the measurements, such as sample preparation, fitting data, thermal stability, and vibration. Random uncertainties were calculated using the standard uncertainty defined as u(T)=s/n, where s represents the standard deviation and n represents the number of repetitions. In our situation, n was 4. The uncertainty in the temperature was define by the following equation:

$$u(T)^2 = u(T)^2 + u_R(T)^2$$

The uncertainty in the enthalpies depends on the heat flow, Q, sample mass, m, and temperature, T. Random uncertainties also impact the measurements, such as sample preparation, impurities, fitting data, and thermal stability. Random uncertainties were calculated using the standard uncertainty defined as u(T)=s/n, where s represents the standard deviation and n represents the number of repetitions. In our situation, n was 4. The uncertainty in the enthalpy was define by the following equation:

$$u(h)^2 = \left(\frac{dh}{dQ}\right)^2 u(Q)^2 + \left(\frac{dh}{dm}\right)^2 u(m)^2 + \left(\frac{dh}{dT}\right)^2 u(T)^2 + u_R(h)^2$$

1.6 Thermal Conductivity

Calibration. Both sensors were calibrated before measurements using a single point technique. The reference material used for the calibration was Polymethyl Methacrylate (PMMA) with a thermal conductivity of 0.193 W/mK at 293 K. Furthermore, the thermal conductivity, λ of distilled water was measured with the THB6K/MFR sensor to verify that the calibration was successful. The thermal conductivity of water is 0.598 W·mK^{-1} at 293 K[28].

Solid sample preparation. the materials were melted at about T_m + 5 K, and then was poured into a previously heated metallic mould, as shown in Figure 2Sa. The metallic mould was heated in a hot plate to a temperature about T_m - 5 K. Once the mold was filled, the temperature was reduced to room temperature approximated at 1 K min^{-1}. This method proportioned a smooth surface on the sample, improving the contact with the THB6K sensor. The dimensions of the solid samples were 50 x 30 x 8 mm (Figure 2Sb). Six samples (three pairs) were prepared using this procedure for each material.

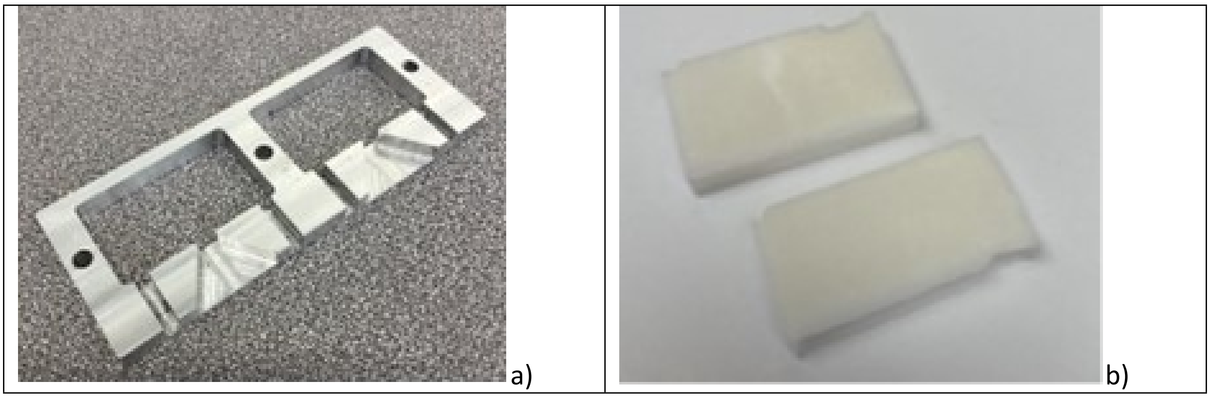

Figure 2S. Thermal conductivity : a) metallic mould and b) solid samples

The uncertainty of the thermal conductivity was due to direct measure of the thermal conductivity that according to manufacture of the THB-100 equipment is better than 3 %. There are also random uncertainties that affect the thermal conductivity measurements such the smooth of the surfaces, vibration, skills of the operator, sample preparation, purity of material. The ramdon uncertainties were calculated using the standard uncertainty that is define as $u_R(\lambda) = s/\sqrt{n}$, where s is the standard deviation and n is the number of repetitions. Then, temperature fluctuations also affect to uncertainty of the thermal conductivity.

According with the guidelines described in the GUM, the thermal conductivity combine uncertainties can be calculated with the following equation:

$$u(\lambda)^2 = u(\lambda)^2 + u_R(\lambda)^2 + \left(\frac{d\lambda}{dT}\right)^2 u(T)^2$$

Where $u(\lambda)$ is the uncertainty provide by manufacture, $u_R(\lambda)=s/\sqrt{n}$ is the standard uncertainty due to random error such as sample preparation and $u(T)$ uncertainty due to the temperature.

2. Thermogram from hf-DSC

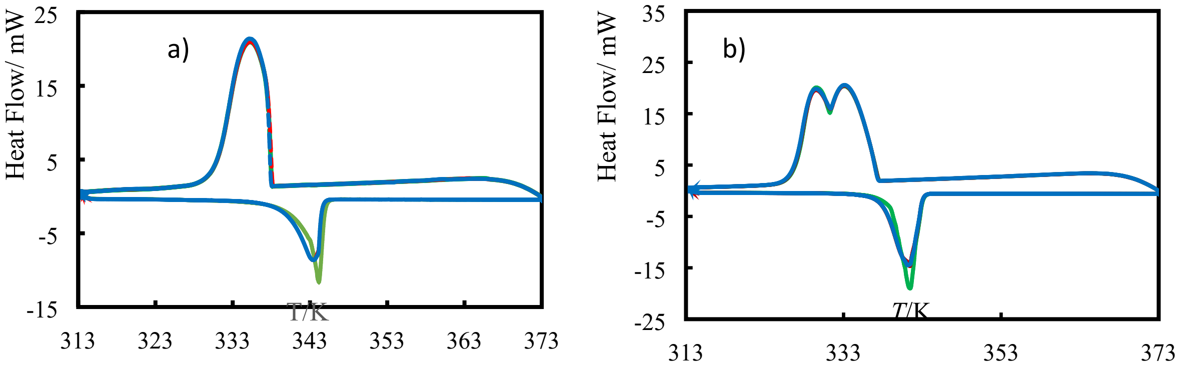

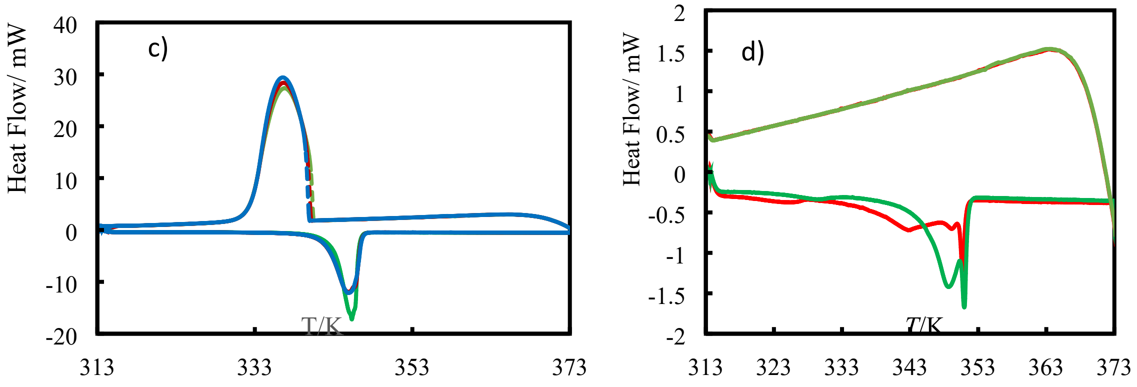

Figure S3. Heat flow versus temperature at 0.5 K·min^{-1} obtained by hf-DSC for a) OM65; b) RT64HC; c) Stearic acid; d) [N_{4444}^{+}][Cl^{-}] for three cycles: cycle 1, cycle 2 and cycle 3.

3. Properties measurements

Viscosity is an essential transport property for thermal energy applications; it is required to design equipment such as pumping systems and analyse the effectiveness of the fluid for heat and mass transfer processes. Phase change properties, such as melting, and solidification temperatures of ionic liquids are related to its application as PCM for latent heat thermal energy storage (LHTES). The thermal conductivity is essential for the heat transfer process The cooling and heating efficiency and the energy transferred depend highly on this property. In general, lower levels of thermal conductivity often provide better heat retention, while higher values allow more efficient heat transport.
